# Releasing the Brake: Targeting Cbl-b to Enhance Lymphocyte Effector Functions

**DOI:** 10.1155/2012/692639

**Published:** 2012-04-10

**Authors:** Stephanie Wallner, Thomas Gruber, Gottfried Baier, Dominik Wolf

**Affiliations:** ^1^Department of Internal Medicine V—Hematology and Oncology, Medical University Innsbruck, 6020 Innsbruck, Austria; ^2^Department of Medical Genetics, Molecular and Clinical Pharmacology, Medical University of Innsbruck, Innsbruck, Austria; ^3^Department of Internal Medicine III, Hematology and Oncology, University Hospital Bonn, Sigmund-Freud Strasse 25, 53127 Bonn, Germany

## Abstract

The E3 ubiquitin ligase Cbl-b is an established nonredundant negative regulator of T-cell activation. Cbl-b fine-tunes the activation threshold of T cells and uncouples T cells from their vital need of a costimulatory signal to mount a productive immune response. Accordingly, mice deficient in *cblb* are prone to autoimmunity and reject tumors. The latter has been described to be mediated *via* CD8^+^ T cells, which are hyperactive and more abundant in shrinking tumors of *cblb*-deficient animals. This might at least also in part be mediated by resistance of *cblb*-deficient T cells to negative cues exerted by tumor-associated immuno-suppressive factors, such as TGF-**β** and regulatory T cells (Treg). Experiments using *cblb*-deficient T cells either alone or in combination with vaccines validate the therapeutic concept of enhancing the efficacy of adoptively transferred lymphocytes to treat malignant tumors. This paper summarizes the current knowledge about the negative regulatory role of Cbl-b in T-cell activation and its potential therapeutic implications for cancer immunotherapy.

## 1. Introduction

**Figure 1:**
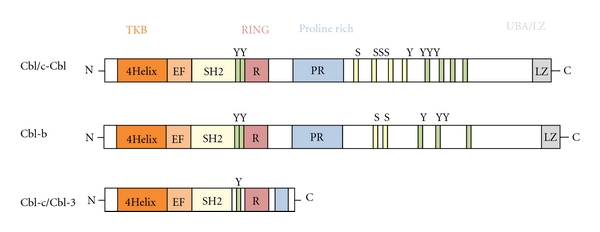
The mammalian Cbl protein family. Schematic representation of the domain architecture of the three mammalian Cbl isoforms, c-Cbl, Cbl-b, and Cbl-c/Cbl-3. The Cbl proteins are highly conserved in the N-terminal region where they comprise a tyrosine-kinase-binding domain (TKB), which is composed of a 4-helix bundle (4H), a calcium-binding EF domain, and a variant SH2 domain that is linked with the RING finger domain. The COOH-terminal region contains proline-rich stretches, multiple serine and tyrosine phosphorylation sites and a ubiquitin-associated UBA domain, and a leucine zipper. The Cbl-c isoform lacks the UBA domain and the leucine zipper domain. TKB, tyrosine-kinase-binding domain; 4H, four-helix bundle; EF, EF hand; SH2, Src-homology domain 2; R, RING “really interesting new gene” finger domain; PR, proline rich domain; Y, tyrosine residue; S, serine residue; LZ/UBA, leucine zipper/ubiquitin associated domain.

Maintenance of tolerance and induction of T-cell anergy is critical for prevention of autoimmunity. However, in the case of malignancies, tumor-induced T-cell anergy and/or tolerization induces cancer-associated immune paralysis, which at least in part contributes to uncontrolled tumor growth and metastasis. In 2000, two groups independently described that the E3 ligase Cbl-b functions as a “gate keeper” in peripheral T-cell tolerance [1, 2]. Cbl-b is a member of the highly conserved family of Cbl (casitas b-lineage lymphoma) proteins (Figure 1) and ubiquitinylates substrates by its E3 ligase activity via its RING domain. The name is derived from the retroviral oncoprotein v-Cbl, which promotes development of b-cell lymphoma in mice [3]. Target ubiquitinylation induces either proteasomal or lysosomal degradation regulating protein turnover or modifies the function of target proteins by altered subcellular localization. Cytoplasmic signaling proteins and nuclear transcription factors tend to be polyubiquitinated for subsequent proteasomal degradation, whereas ligand-activated surface receptors, such as receptor tyrosine kinases, G-protein-coupled receptors, and the T-cell receptor (TCR) are more often regulated by endocytosis followed by lysosomal degradation [4–6]. Substrates can either be tagged with single (monoubiquitinylation) or multiple (>4) ubiquitins, the latter leading to polyubiquitinylation. These polyubiquitin chains are generally linked by lysine residues at position 48 or 63. It is known that polyubiquitintagging of substrates induces their degradation by the 26S proteasome, but polyubiquitin chains might also modify protein function, for example, by increased cell-surface-receptor turnover or an altered subcellular localization. In contrast, monoubiquitinylation has been shown to target cell-membrane-receptor-associated proteins to the lysosome, thereby attenuating cell-surface-receptor-mediated signals by a desensitization process. Thus, monoubiquitinylation and K63-linked polyubiquitinylation rather regulate protein trafficking and cell-cell interactions, whereas K48-linked polyubiquitinylation targets substrates to the proteasome for degradation [7]. Cbl-b is critically involved in these ubiquitinylation processes, and due to its preferential expression in immune cells it is centrally involved in the regulation of immune responses. In more detail, deficiency of Cbl-b in T-cells induces a prominent hyperactive phenotype, resulting in systemic signs of autoimmunity in animals lacking a functional *cblb* gene [1, 2]. It is tempting to speculate that inhibition of its function might also represent a rational approach to increase T-cell reactivity in the cancer setting. *Vice versa*, in the case of an exaggerated immune response, such as seen in autoimmunity and graft rejections, induction of Cbl-b might be an attractive strategy to limit T-cell reactivity finally inducing tolerance.

This review will summarize our current knowledge on the role of Cbl-b as regulator of T-cell effector function with a particular focus on its potential therapeutic use as target in cancer immunotherapy.

## 2. Cbl-b is a Member of the Cbl Family of Proteins

Cbl-b is a member of a highly conserved family of Cbl proteins, which in mammals consists of three Cbl genes: Cbl (also termed c-Cbl, Cbl2,or RNF55), Cbl-b (also termed RNF56), and Cbl-c/Cbl-3 (also termed Cbl-SL or RNF57) (Figure 1) [8]. Cbl proteins interact with target proteins via their protein-protein interaction domains, allowing regulation of multiple signaling pathways [9, 10]. The E3 ligase activity of c-Cbl as well as the other Cbl proteins has been shown to depend on the RING-type zinc finger domain [11, 12]. Data from *in vitro *experiments using deletion mutations of c-Cbl highlight the critical role of the RING finger domain for its E3 ligase activity, as RING finger domain mutants could not ubiquitinylate the EGF receptor [13, 14] with the consequence that ligand-induced c-Cbl-mediated desensitization of EGFR receptor expressing cells to EGF was disrupted. The RING finger domain also binds the E2 conjugating enzyme and mediates transfer of ubiquitin between E2 and target substrates [11]. Loss of function mutations in the E3-ligase domain of* cblb *in mice phenocopies the *cblb*-knockout phenotype [15], again supporting the important role of the E3 ubiquitin ligase activity for Cbl-b function. 

## 3. Cbl-b as Master Regulator of T-Cell Effector Functions

Following initial triggering of the antigen receptor, the Src family kinases Lck and Fyn are recruited and phosphorylate Zap-70 (zeta-associated protein of 70 kDa), which initiates various downstream signaling pathways [16]. ZAP-70 phosphorylates SLP-76 mediating formation of a multisubunit protein complex including key signaling molecules such as phosphatidylinositol 3-kinase (PI3K), PLC (phospholipase C), and Vav1. Activation of these signaling components results in the activation of PLCy-regulated Ca^++^ influx, cytoskeletal rearrangements via the nucleotide exchange factor Vav1, Rac,and WASP, and activation of the protein kinase C*θ* (PKC*θ*) [17–19]. The activation step of PKC*θ* is essential for an appropriate NF*κ*B stimulation to induce a productive T-cell immune response *in vivo*. Of note, PKC*θ* also functions as critical intermediary for inactivation of Cbl-b in response to costimulation of T-cells through CD28 [20].

**Figure 2:**
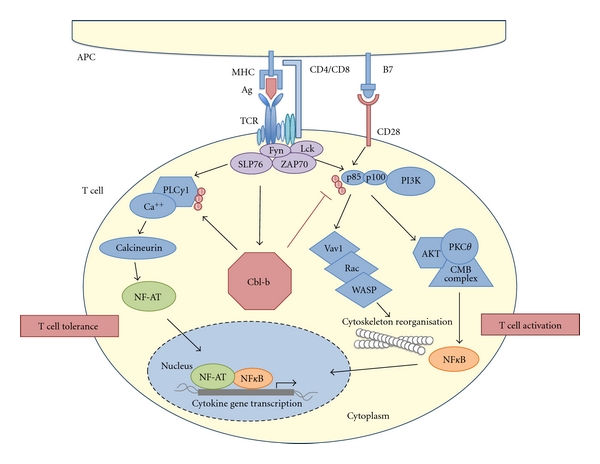
Cbl-b functions as central gate keeper of T-cell activation. T-cell stimulation via the TCR and the second costimulatory signal leads to the proximal activation of signaling pathway components. In the absence of costimulation, Cbl-b promotes antigen-specific T-cell tolerance. Thus, Cbl-b functions as negative regulator of the activation of T cells that can be, however, bypassed by CD28 stimulation. In the absence of Cbl-b, T cells are not dependent on a costimulatory signal and proliferative as well as cytokine response upon sole TCR-activation is comparable to WT T cell stimulated via the TCR and CD28. Note that the signaling cascades and interactions are simplified and do not show all molecules involved. For further details please refer to text. Flat-ended lines indicate inhibitory interactions. APC, antigen—presenting cell; U, Ubiquitin.

In a physiological context, Cbl-b apparently acts as a negative regulator of the T-cell activation process (Figure 2). Accordingly, *cblb*-deficient mice are highly susceptible to spontaneous and antigen-induced experimental autoimmune diseases [1, 2], despite normal thymic T-cell selection and normal peripheral T cell numbers. When isolated, *cblb*-deficient T cells are hyperproliferative and bypass the requirement for CD28 costimulation, that is, they proliferate upon sole stimulation via the TCR comparable to WT T cells stimulated with both anti-CD3 and anti-CD28 stimulating antibodies [1, 2]. Moreover, they produce markedly higher levels of proinflammatory cytokines, such as IFN-*γ* [21]. Accordingly, T-cell anergy promoting conditions, such as TCR stimulation in the absence of CD28 costimulation, induce Cbl-b expression [22]. 

The TCR induces Ca^++^ influx and subsequent activation of the transcription factor NFAT, which leads to activation of the early growth response genes Egr-2 and Egr-3, which then increase Cbl-b expression [4, 23]. Cbl-b in turn regulates recruitment of p85 to CD28 and the TCR zeta chain through its E3 ubiquitin ligase activity. The observation that PI3K inhibition reverts enhanced activation of *cblb*-deficient T-cells supports the importance of PI3K as Cbl-b target (see Figure 2) [24]. As a consequence of these molecular interactions, induction of anergy is prevented in T cells deficient in Cbl-b *in vitro* and *in vivo* [22]. As an example for the *in vivo* relevance of Cbl-b in anergy induction, severe collagen-induced arthritis can be induced in *cblb*-deficient mice even in the absence of the adjuvant, again highlighting the hyperactive state of the T cell compartment in these animals [22]. Moreover, in a model of anergy induction in T cells, which makes use of mice carrying the p14 TCRVa2Vb8.2 transgene recognizing the lymphocytic choriomeningitis virus (LCMV) p33 peptide presented by MHC class I, the animals were challenged by repeated injections of the cognate peptide p33. Of note, in this model, *cblb*-deficient p14 transgenic mice challenged with p33 massively activated CD8^+^ T cells. This induced significant mortality mediated by a severe cytokine storm [22]. When p14/Rip-gp transgenic mice are exposed to LCMV, the animals develop diabetes. The rate of animals developing disease, however, is substantially lower when p14/Rip-gp transgenic mice are challenged with the low agonistic peptide LCMV-LF6 (diabetes rate <50%), whereas injection of *cblb*-deficient animals with this low agonistic peptide induced rapid onset of diabetes paralleled by an enhanced CTL function [25]. From a mechanistic perspective, Cbl-b reduces phosphorylation of PLC-*γ*1 resulting in reduced PLC-*γ*1 activity in anergic T cells [22]. Moreover, in addition to an intrinsic hyperactive phenotype, *cblb* deficiency also induces resistance towards negative cues from the environment, such as soluble TGF-*β* [26] or effector immune cell suppression mediated by Treg [27]. This is also reflected by decreased generation of inducible Treg in naive *cblb*-deficient T cells, as this process is also mediated by TGF-*β* [28]. Interestingly, in this particular model the TGF-*β* pathway was not shown to be defective, but impaired FoxP3 induction was mediated by increased phosphorylation of Foxo3a/Foxo1 in *cblb*-deficient naive CD4^+^ T cells. In contrast, partial TGF-*β* resistance has been well documented in CD4^+^ T cells, which have reduced levels of phosphorylated SMAD2 upon TGF-*β* stimulation [26]. Thus, Cbl-b clearly interacts with the TGF-*β* signaling pathway, but the exact molecular background for this observation remains controversial. Nonetheless, resistance of *cblb*-deficient T cells to these negative environmental factors might at least in part explain their increased antitumor efficacy (see below).

## 4. Cbl-b as Potential Target in Cancer Immunotherapy

Various reports now linked Cbl-b with anticancer immune responses. First, Loeser and colleagues demonstrated that* cblb*-deficient animals are less susceptible to tumor formation in induced as well as spontaneous mouse cancer models relevant for human cancers [29]. Subcutaneous implantation of TC1 tumor cells as well as induction of spontaneous tumors by UV irradiation in *cblb*-deficient animals led to a significantly delayed outgrowth of tumors, when compared to WT animals. Of note, although almost 100% of *cblb*-deficient animals had a delayed tumor growth, only a few animals completely rejected the tumor. It remains to be determined which variables determine complete rejection versus delayed tumor growth. However, tumor rejection was paralleled by an increased infiltration of CD3^+^CD8^+^ T cells into the tumor. Depletion experiments corroborated the functional importance of this particular cell population in tumor rejection, because CD8-depleted animals showed tumor growth comparable to WT animals. In addition, increased tumor infiltration of CD8^+^ T cells also led to markedly increased amounts of the proinflammatory cytokine IFN-*γ* within the tumor microenvironment, reflecting the boosted immune response *in vivo*. Finally this led to an impaired tumor cell proliferation as quantified by Ki67 staining and an increased rate of apoptosis, as determined by caspase-3 detection. The same group supported this concept by reproducing these results in mice lacking a functional E3 ligase domain due to a loss-of-function mutation within this region of the *cblb* gene. Again, mice that rejected TC1 tumors had increased CD8^+^ T cells infiltrating tumors leading to reduced proliferation and increased apoptosis [15]. These data have been further supported by the observation that *cblb*-deficient animals also reject EL4 and EG7 tumor cells [30] as well as spontaneous tumors generated by crossing *cblb*-deficient with ataxia telangiectasia mutant (ATM) mice, which normally develop thymic T-cell lymphoma. Interestingly, these authors also provided genetic evidence that the lack of costimulation is also of *in vivo* relevance, as *cblb-* and CD28-deficient mutant mice are protected against EL4 cells as well. Thus, these mice can generate a productive antitumor immue response even in the absence of CD28 [30]. However, when using an adoptive transfer model, the data are somehow contradictory. First, Chiang and colleagues described that adoptive transfer of 3 × 10^6^ polyclonal *cblb*-deficient CD8^+^ T cells are hyperactive and reject at least in part EG7 tumors. Moreover, overall survival in this particular model was significantly increased in animals receiving *cblb*-deficient versus WT CD8^+^ T cells. In contrast, we were not able to detect any protective effect of 5 × 10^6^ polyclonal CD8^+^ T cells neither in the B16-Ova nor in the EG7 tumor model. We hypothesized that a second *in vivo* activation would be necessary, which allows efficient activation and selection of tumor model antigen positive T cells. Thus, we combined the adoptive T-cell transfer with the application of a dendritic cell (DC) vaccine, which now induced profound antitumor immune effects *in vivo*, which were paralleled by expansion of Ova-specific CD8^+^ T cells, higher infiltration of CD8^+^ T cells into the tumor and higher expression of IFN-*γ* [21]. This observation is also supported by data from Yang and colleagues, who showed that *cblb*-deficient CD8^+^ T cells are indeed generally more efficacious but are nonetheless unable to mediate curative responses against all tumor types [31]. Using various tumor models expressing an ovalbumin-tagged version of HER-2/*neu *receiving adoptive transfer with WT or* cblb*-deficient CD8^+^ T cells from OT-I T-cell receptor transgenic donor mice, the authors could demonstrate that at least some tumors (e.g., NOP18) could not be rejected due to insufficient infiltration of Ova-specific T cells into the tumor microenvironment. These data highlight the need of proper migration of tumor-reactive T cells into the tumor microenvironment to attack cancer cells. If this infiltration is prevented, even hyperactive *cblb*-deficient T cells are not able to kill malignant cells.

## 5. Summary and Perspective

Data from knockout animal studies suggest that inactivation of Cbl-b, which is a nonredundant negative regulator of effector CD8^+^ and CD4^+^ T-cell signaling, represents a rational approach to improve anticancer T-cell reactivity *in vivo*. Proof-of-concept studies using adoptive transfer of isolated hyperactive T cells from *cblb*-deficient animals in tumor models validate Cbl-b as potential therapeutic target for improvement of antitumor immunotherapy. Future studies will help to identify strategies allowing *in vivo* pharmacological targeting of Cbl-b activity or genetic modification of Cbl-b expression in adoptively transferred T cells (e.g., by siRNA).
